# Preparation and DFT Study for New Three-Ring Supramolecular H-Bonded Induced Liquid Crystal Complexes

**DOI:** 10.3389/fchem.2021.679528

**Published:** 2021-06-04

**Authors:** Sayed Z. Mohammady, Daifallah M. Aldhayan, Mohamed Hagar

**Affiliations:** ^1^ Chemistry Department, Faculty of Science, King Saud University, Riyadh, Saudi Arabia; ^2^ Chemistry Department, Faculty of Science, Cairo University, Giza, Egypt; ^3^ Chemistry Department, College of Sciences, Taibah University, Yanbu, Saudi Arabia; ^4^ Chemistry Department, Faculty of Science, Alexandria University, Alexandria, Egypt

**Keywords:** supramolecular, liquid crystal complexes, Schiff base, DFT, mesomorphic transitions

## Abstract

Supramolecular three-ring Schiff base novel liquid crystal complexes have been prepared and investigated. Schiff bases of para-substituted aniline derivatives and para-pyridine carbaldehyde have been prepared and then mixed in equimolar quantities with para-alkoxy benzoic acids. On one side, the alkoxy chain length varies from 8 to 16 carbon atoms. On the other side, terminal small compact groups substituting aniline with various polarities are used. Hydrogen-bonding interaction was elucidated by FTIR spectroscopy. The mesomorphic thermal and optical characteristics of the samples were obtained by differential scanning calorimetry (DSC) and polarized optical microscopy (POM). All samples exhibit enantiotropic mesophases. Experimental results obtained for the induced mesophases were correlated with density functional theory (DFT) theoretical calculations. The results revealed that both the polar compact groups’ polarity and the alkoxy chain lengths contribute strongly to mesomorphic characteristics and thermal stabilities of the mesophases. Surprisingly, the observed values of enthalpy changes associated with the crystalline mesomorphic transitions lie in the range of 2.2–12.5 kJ/mol. However, the enthalpy changes corresponding to the mesomorphic–isotropic transitions vary from 0.9 to 13.9 kJ/mol, depending on the polarity of para-attached groups to the aniline moiety.

## Introduction

The supramolecular aggregation arises from the binding of a group of molecules of a well-defined structure. These molecules are grouped together by second-order (noncovalent) bonds, for example, hydrogen bonds, halogen bonds, pi agglutination, van der Waals forces, coordination bonds, and dipole–dipole interactions (Borissova et al., 2008) (Arunan et al., 2011) (Shimizu and Ferreira Da Silva, 2018). Liquid crystals (LCs) are considered among the most important formative materials that can be produced by supramolecular assembly (Park et al., 2015) (Sun et al., 2015) (Alnoman et al., 2020) (Scholte et al., 2020). The created assemblies in liquid crystals have been used for diverse applications (Chen et al., 2013) (Chen et al., 2014) (Todisco et al., 2018) (Guan et al., 2019) (Foelen et al., 2020).

Among the second-order bonding types, hydrogen bonds play a pivotal role in supramolecular assemblies, and this has resulted in their widespread use for the synthesis of LCs. The most common method of preparing two-component self-assembly is mixing followed by two-component reaction through hydrogen bonding either in the melt or from solutions (Arikainen et al., 2000) (Lozman et al., 2001) (Watt et al., 2004).

In addition, hydrogen bonding has been applied to a wide range of academic research (Voutsas et al., 2000) (Crisp and Jiang, 2002) (Thote and Gupta, 2003) and to systems of industrial importance (Lammers, 1991) (Thote and Gupta, 2003). To name just a few, it has a recent application in improving the performance of liquid crystal displays (LCDs), with hydrogen bonding arising between the liquid crystal and the dichroic dye dissolved in it (Kataoka et al., 2000) (Matsude, 2000) (Thote and Gupta, 2003). Liquid crystal displays are one of the major applications of liquid crystal in the industry. LCDs are basically two panels with perpendicular polarizations. The liquid crystal is the active participant between the two panels and is characterized by a dichroic dye dissolved in them.

Differential scanning calorimetry (DSC) results of liquid crystals even for those produced through supramolecular assemblies usually possess relatively high enthalpy changes (**ΔH**) accompanied with the crystalline mesomorphic transitions (∼20–100 kJ/mol) (Tsuji et al., 1979) (Yousif and Al-Hamdani, 1993) (Domalski and Hearing, 1996) (Oweimreen and Morsy, 1999) (2000) (Chidichimo et al., 2004) (Acree and Chickos, 2006). However, few examples have been reported in the literature where the **ΔH** values of melting are lower than 10 kJ/mol (Du et al., 2019) (Cramer et al., 2020) (Huang et al., 2020) (Wang et al., 2020).

In this work, new supramolecular three-ring Schiff base–induced liquid crystal complexes will be prepared and investigated. Schiff bases of para-substituted aniline derivatives and para-pyridine carbaldehyde have been prepared and then mixed in equimolar quantities with para-alkoxy benzoic acids. The alkoxy chain length varies from 8 to 16 carbon atoms, namely, C8, C10, C12, and C16. On the other side, terminal small compact groups substituting aniline with various polarities are used, namely, the methoxy, methyl, chloro, bromo, nitro, and flouro groups. Hydrogen-bonding interaction is expected to take place to produce three ring–induced hydrogen-bonded liquid crystals. The alkoxy acid is the hydrogen-bond source, while the pyridine structural units present the hydrogen-bond acceptor. The mesomorphic thermal and optical characteristics of the samples will be investigated by DSC and polarized optical microscopy (POM). It is worth noting that, generally, the system under investigation possesses extremely low crystalline mesomorphic **ΔH** changes (reaches ∼2.0 kJ/mol). Finally, pursuing our interest (Al-Mutabagani et al., 2020) (Hagar et al., 2020) (Nafee et al., 2020) (Ali et al., 2021) (Almehmadi et al., 2021) (Mohammed et al., 2021) (Parveen et al., 2021) in conducting the experimental results with density functional theory (DFT) theoretical calculations is another goal.

## Materials and Methods

**SCHEME 1:**
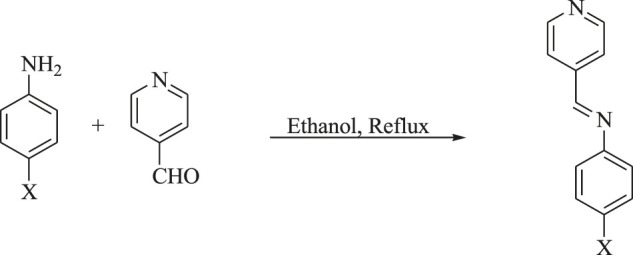
Schiff bases A–F preparation.

**SCHEME 2:**
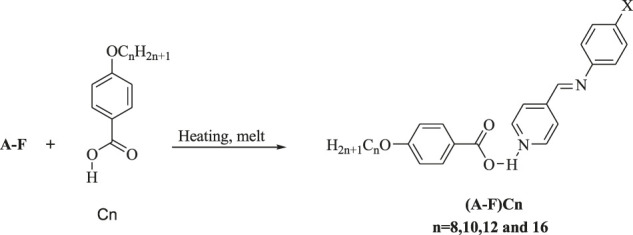
Preparation of the complexes [(A–F)Cn].

All chemicals were purchased from TCI Company, Japan. Their purity is higher than 98%. Schiff bases were prepared and recrystallized twice from ethanol–water mixture and were checked to be TLC pure (see Scheme 1). Finally, supramolecular complexes were prepared by mixing equimolar ratios of a particular Schiff base with the para-alkoxy benzoic acid and then subjected to melting to ensure the formation of the complexes (see Scheme 2). The compounds were identified by their reported melting points: A (98 C) (Boyer et al., 2007), B (100 C) (Poronik et al., 2017), C (88 C) (Kouznetsov et al., 2017), D (74 C) (Krishna Murthy et al., 2018), E (80 C) (Boyer et al., 2007), and F (82 C).

Fourier-transform infrared spectroscopy (FTIR) spectra were recorded on a spectrometer (Shimadzu, model: IRAffinity, Japan). Samples were prepared as KBr pellets.

The phase changes in the materials were determined via differential scanning calorimetry (DSC). Japan's Shimadzu DSC-60A was used. 2–3 mg specimens were encapsulated in aluminum pans and heated or cooled in a dry nitrogen setting. The samples were heated at 10.0°C/min during all the heating and cooling curves of all samples. The samples were heated to 150°C from room temperature and then cooled to 10°C. Finally, the samples were reheated to 200°C at the same heating rate under an inert atmosphere of nitrogen gas. The accuracy in temperature monitoring is lower than 1.0°C.

## Results and Discussion

### FT-IR Characterizations

**FIGURE 1:**
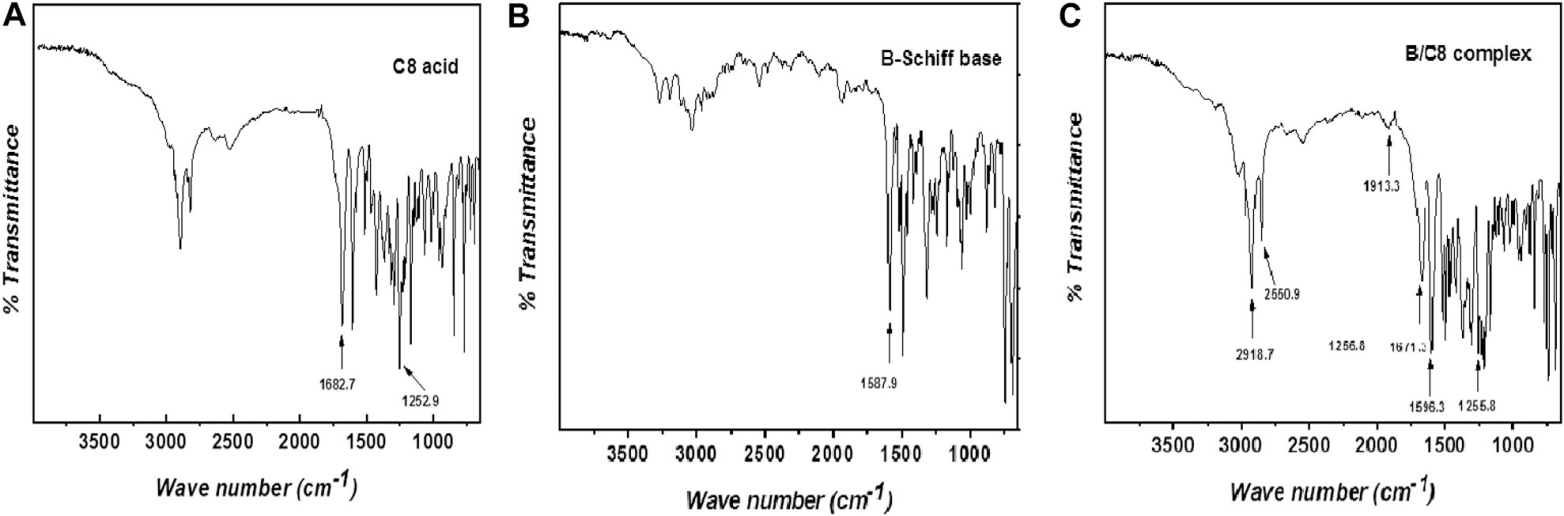
**(A–C)**. FT-IR spectra of para-octyloxy benzoic acid (C8), the methyl Schiff base **(B)**, and the complex B/C8 in charts **A–C**, respectively.

Condensation of 4-pyridine carbaldehyde with arylamines yielded the nonmesomorphic nitrogen-based portion. The Schiff bases were used to prepare supramolecular H-bonded complexes {[(A-F)Cn]}. The particular Schiff base was mixed in 1:1 M ratios with the corresponding alkoxy benzoic acid at particular chain lengths (*n* = 8, 10, 12, and 16). The formation of the complexes was proved *via* FT-IR and NMR spectral analysis (Saunders and Hyne, 1958) (Lam et al., 2016) (Martinez-Felipe et al., 2016) (Hu et al., 2017) (Pothoczki et al., 2021). However, FT-IR measurements proved to be an effective tool for such confirmation (Martínez-Felipe and Imrie, 2015) (Paterson et al., 2015) (Martinez-Felipe et al., 2016) (Alhaddad et al., 2020) (Sherif et al., 2020). The measurements of the spectral data were measured for the individual compounds B, C8, and B/C8 and are given in Figures 1A–C **,** where A, C8, and B/C8 represent the methyl Schiff base, p-octyloxybenzoic acid, and the complex.

The existence of the prepared H-bonding complexes has been proved by FTIR (Figure 1 A–C). The measurements were carried out for the free acids (Cn), Schiff bases (A–F), and for their corresponding supramolecular complexes [(A–F)Cn].

Figure 1A shows a signal at 1,682.8 cm^−1^ attributed to the C=O group of the free alkoxy acid. The H-bonds between the nitrogen atom of the pyridine Schiff base portion (B) and the hydrogen donor para-octyloxy benzoic acid (C8) of the supramolecular complex (B/C8) shift the position of the stretching vibrations of the C=O group from 1,682.8 to 1,671.3 cm^−1^, which is strong evidence of the H-bonding in supramolecular formed complexes. This pronounced shift of wave number values (lowered by 11.5 cm^−1^) implies that the formation of the B/C8 complex has an intensively negative effect on the C=O ester of the acid.

The FTIR results are extended further to elucidate the formation of the H-bonded complexes through the existence of the three Fermi resonance vibrational bands associated with H-bonding formation in the complexes. The vibrational bands assigned appearing at 2,918.7, 2,850.9, and 1,913.0 cm^−1^ (Figure 1C) can be rationalized to A-type, B-type, and C-type Fermi bands, respectively. The appearance of the A-type band is evidence of the involvment of the OH group in H-bonding (Babkov et al., 2012). In addition, the band revealed at 2,850.9 cm^−1^ (Figure 1C) could be rationalized to the O–H in-plane bending vibration and its associated fundamental stretch (type B). The overtone of the torsional effect and the OH fundamental stretching vibration causes the third band (C-type Fermi band) to appear at 1,900 cm^−1^.

There is an observed shift in the peak maximum of the etheric C–O bond in the acid upon complex formation. The C–O peak maximum of the acid is revealed at 1,252.9 cm^−1^ (Figure 1A), while it is observed at 1,255.9 cm^−1^ in the complex (Figure 1C). In addition, there is a clear shift of wave number values corresponding to the –CH=N– Schiff base main peak from 1,587.9 cm^−1^ (Figure 1B) to 1,596.3 cm^−1^ in the complex (Figure 1C). This increase in the peak position value of the azomethine group (8.6 cm^−1^) can be extra evidence for the formation of the B/C8 complex.

The three-ring 1:1 complexes [(A-F)Cn] were studied in terms of mesophase and optical analysis. Table 1 summarizes the transition temperatures (T), associated enthalpies (H), and normalized entropies (S/R) of all mesophase transitions, as calculated by DSC measurements, for all prepared [(A–F)Cn] complexes.

**TABLE 1:** Phase transitions: temperatures (*T*,iC), enthalpies (*∆H*, kJ/mol), normalized entropies (*∆S*/R), and mesomorphic range (*∆T*) for the [(A–F)Cn] complexes.

		°C	kJ/mol	°C	kJ/mol	°C	kJ/mol	°C	kJ/mol	°C	kJ/mol
	Sample	T_Cr-SmA_	ΔH_Cr-SmA_	T_Cr-N_	ΔH_Cr-N_	T_SmA-N_	ΔH_SmA-N_	T_SmA-I_	ΔH_SmA-I_	T_N-I_	ΔH_N-I_
A/C8	C8-MeO			90.4	3.98					128.4	1.73
B/C8	C8-Me			75.4	3.74					94.6	2.43
C/C8	C8-Cl			88.7	4.47					133.8	3.28
D/C8	C8-Br			95.3	3.65					134.5	2.44
E/C8	C8-F	75.5	4.34			97.2	6.70			147.1	5.30
F/C8	C8-NO_2_			75.5	12.45					124.0	13.94
A/C10	C10-MeO			84.6	4.85					119.7	1.91
B/C10	C10-Me			92.1	8.08					112.9	3.29
C/C10	C10-Cl			88.0	4.09					137.4	2.44
D/C10	C10-Br			97.3	5.22					138.9	2.87
E/C10	C10-F	76.0	5.73			116.6	2.85			142.1	3.19
F/C10	C10-NO_2_			87.3	2.27					131.4	3.73
A/C12	C12-MeO			89.7	6.70					124.7	3.27
B/C12	C12-Me			94	6.49					133.7	3.10
C/C12	C12-Cl			93.5	6.00					134.9	4.09
D/C12	C12-Br			100.7	5.93					137.2	3.41
E/C12	C12-F	64.3	2.97			91.5	4.84			115.6	2.32
F/C12	C12-NO_2_			95.3	6.70					135.0	7.53
A/C16	C16-MeO	92.9	3.91			113.7	0.59			122.9	0.87
B/C16	C16-Me	99.7	5.55					113.2	2.21		
C/C16	C16-Cl	100.0	6.17			116.4	1.87			130.9	2.73
D/C16	C16-Br	106.0	4.11			114.4	1.69			131.8	2.57
E/C16	C16-F	93.9	4.61					113.5	3.71		
F/C16	C16-NO_2_	101.6	5.56			120.7	2.60			136.6	5.32

**FIGURE 2:**
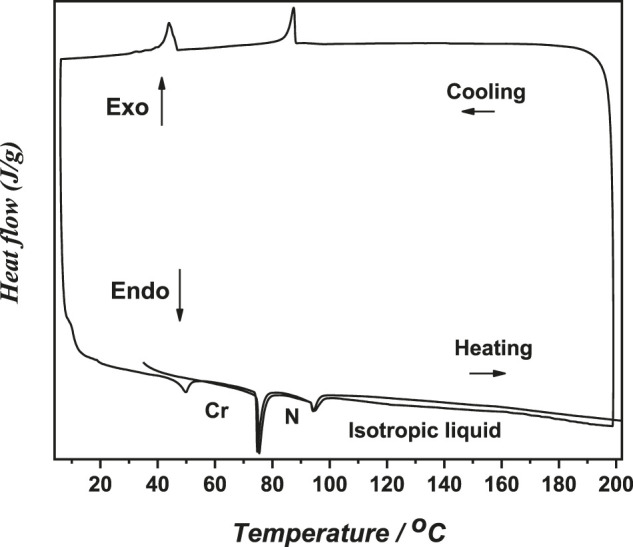
DSC heating/cooling cycles of the B/C8–Me complex liquid crystal sample.

Figure 2 shows the B/C8 differential scanning calorimetric thermograms at a heating rate of 10.0°C/min. We will discuss the results obtained from the second heating run only. The temperature dependence of heat capacity exhibits an endothermic peak at 75.4°C corresponding to a crystalline nematic (Cr-N) phase transition. At elevated temperatures, a second endothermic peak belonging to nematic–isotropic (N-I) transition is observed at 94.6°C.

**FIGURE 3:**
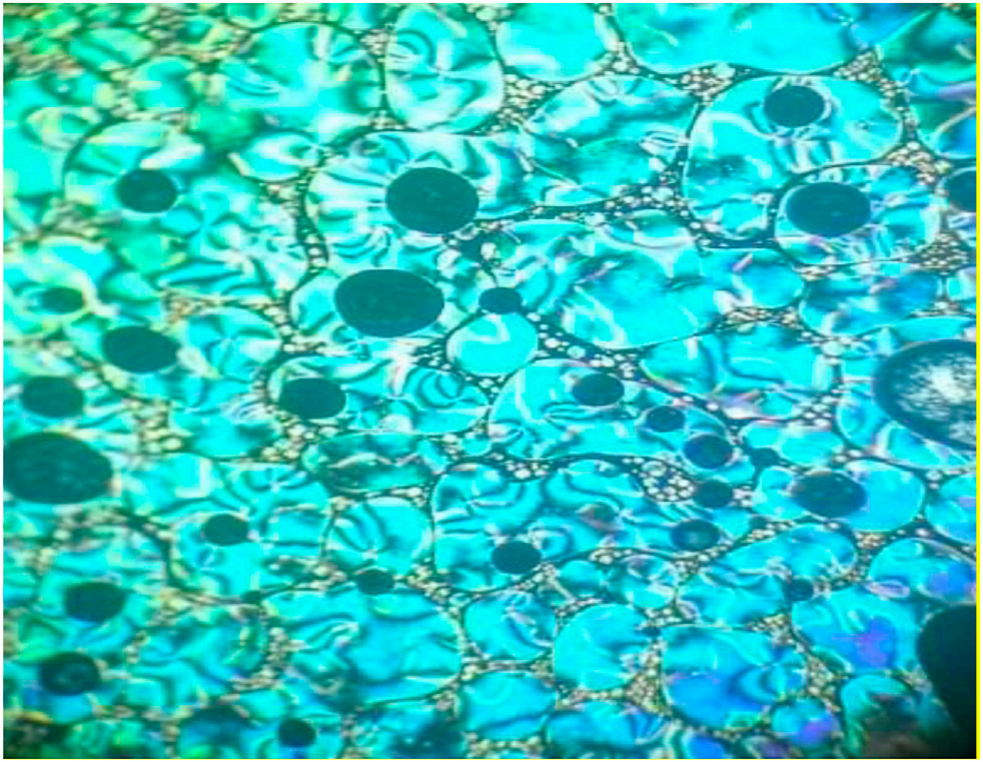
Mesophase textures observed by PLM during the heating cycle of compound A/C8 nematic phase at 101.0 °C.

**FIGURE 4:**
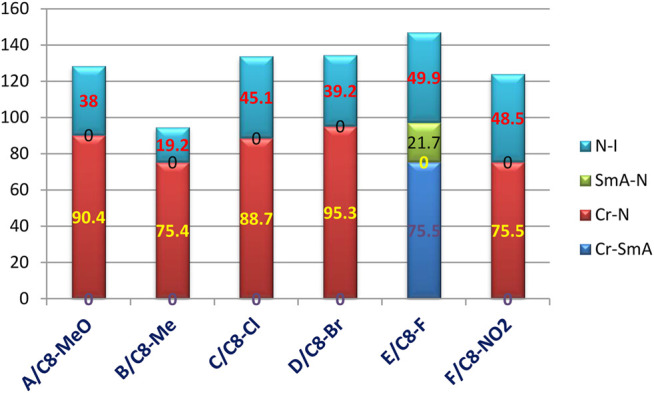
Dependence of the transition temperature on the attached polar group.

As a representative example of the prepared H-bonded supramolecular complexes, an analogous series of C8 of all polar-attached groups are investigated in terms of the transition temperature with respect to the type of the polar group (see Figure 4). The electron-releasing methoxy group showed monomorphic behavior with a nematic range of 38.0°C; however, its analogous methyl group showed a nematic mesophase with a lower nematic range of 19.1°C. This may be explained by the mesomeric resonance effect of the methoxy group's O atom, which raises the π -cloud and allows for more π–π stacking than the methyl group hyperconjugated with the aromatic ring. The comparative investigation of the attachment of the halide atom to the mesomorphic properties is clear in Figure 3. The least electron-withdrawing Br atom enhances the nematic mesophase with a high phase range of 39.2°C; however, the Cl atom showed a longer range of 45.1°C. The higher dipole moment exerted by the stronger negative inductive effect of the chloride group could be explained in terms of the end–end interaction. The incorporation of the compact F atom with the highest electron-withdrawing inductive effect enhances dimorphic mesophases and nematic and smectic A (their ranges were 49.9 and 21.7°C, respectively). The high parallel interaction that could be enhanced due to the attachment of the F atom could be an illustration of the enhanced smectic mesophase and the longer total mesophase range of 71.6°C. The attachment of the nitro group increases the π–π loop to enhance the mesophase range to 48.5°C.

**FIGURE 5:**
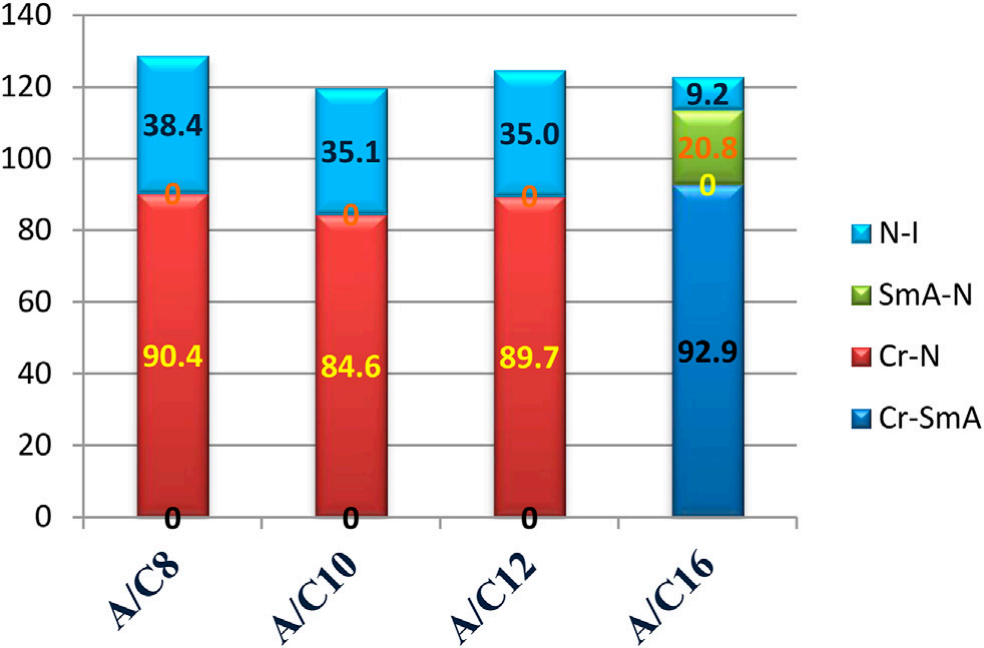
Variation of the transition temperature with the length of the attached alkoxy chain.

The lengths of the attached alkoxy chain, the analogous-bearing terminal methoxy group, and the transition temperatures and their ranges are depicted in Figure 5. The nematic mesophase range decreases as the length of the terminal alkoxy chain increases, with an increase in the appearance of a smectic mesophase for complexes with an alkoxy chain length of 16 carbon atoms at room temperature. Higher terminal aggregation of the alkoxy chain, which promotes higher backing of the molecules, can be used to explain the decrease in the nematic range. The longest chain length, C16, facilitates the highest degree of terminal association to decrease the nematic phase range (9.2°C) and enhances the more ordered smectic mesophase.

### DFT Theoretical Calculations

#### Geometrical Structures

**FIGURE 6:**
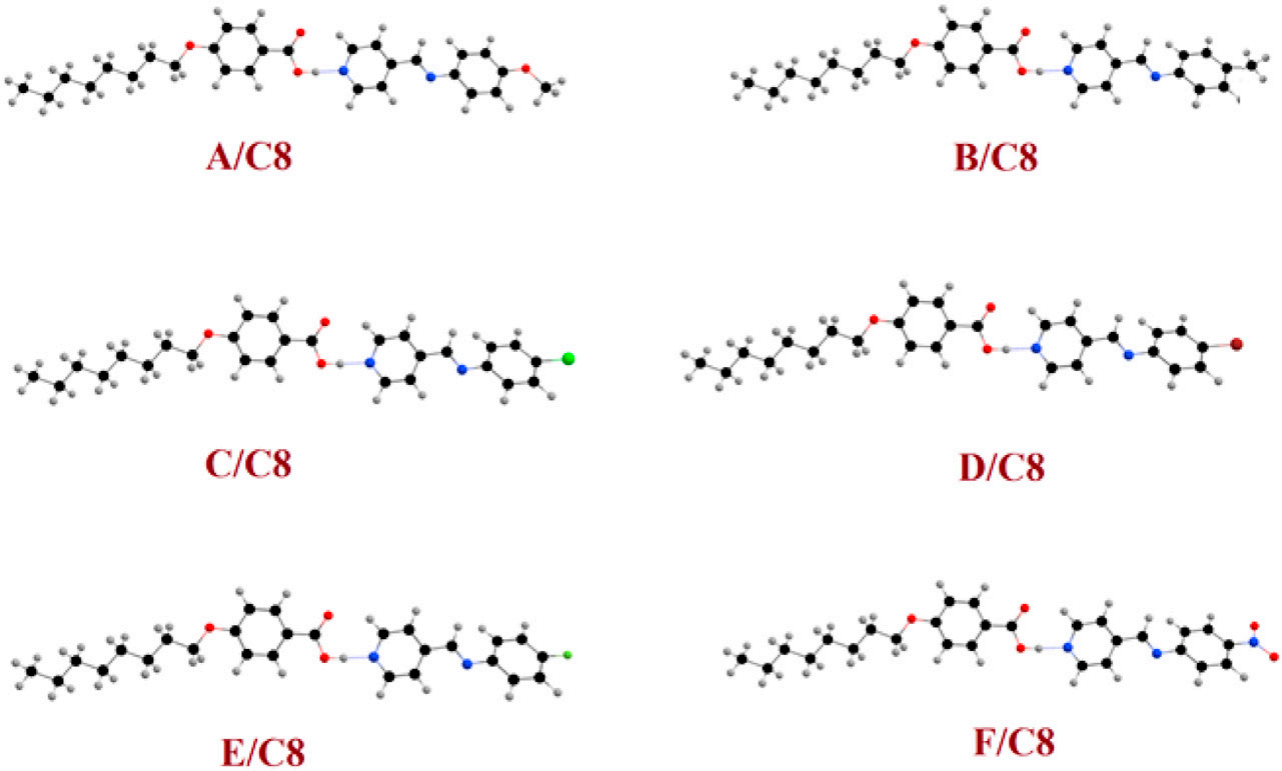
Calculated molecular geometrical structures of the prepared supramolecular complexes of the octyloxybenzoic acid C8 [(A–F)C8].

For all prepared supramolecular complexes of the octyloxybenzoic acid C8 [(A–F)C8], DFT theoretical calculations were performed at the base site B3LYP 6-311g (d, *p*). The calculated optimized geometrical structures of the proposed compounds were calculated in the gas phase using Gaussian 9. All compounds were minimized and optimized and included the estimation of the structural optimization for each compound to find the minimum-energy geometrical structure. The optimization process has been carried out to find the geometrical structure for the minimum energy of conformations, whereas the atoms, the bond lengths, and the bond angle of the compounds demonstrated until a new minimum-energy geometrical structure is established, which is termed as convergence. Then the optimized structures were used in the estimation of the frequency as well as many important thermodynamic parameters. Due to the absence of the imaginary frequency, all optimized molecular structures of all compounds have been found to be stable as shown in Figure 6. The estimated molecular geometry was observed as no co-planar structures with a little bent structure and a twist angle of the Schiff base of the pyridine CH=N bond. The length and the electronic nature of the polar groups and the degree of extra conjugation applied to the structure clearly influence the current twist angle. The least twist angle of the CH=N bond was observed for the nitro derivative θ = 13.1^°^. It could be demonstrated in terms of the nitro group’s extra conjugation, which will ensure the molecules’ planarity. The compact fluorine atom in compound **E/C8**, on the other hand, had the maximum twist angle of 26.7^°^. The bigger the halide atom, the smaller the twist angle, which is 21.1^°^ for chloro and 20.2^°^ for bromo derivatives, respectively. This result could also be interpreted in terms of conjugation, with the larger Br atom less electron-attracting group than the smaller Cl atom one. It should be noted that even though these theoretical estimations of the molecular geometries will offer a good prediction of the favored molecular structure in the gas phase, the presence of these compounds in liquid crystalline matter–condensed phases may exhibit a different lowest energy and more elongated species will be preferred (Paterson et al., 2017). Furthermore, the length of the flexible terminal groups is comprehensively effective on the mesomorphic manner, either the stability or the form of the enhanced phase of liquid crystals, and this is most often accounted for in terms of molecular shape (Hagar et al., 2019).

The electronic properties of the attached substituents are well known to play an important role in the stabilization of one geometrical structure of supramolecular H-bonded complexes than that of the other, where the polar nature of such groups could affect the strength of the intramolecular H-bonding. The effect of a polar substituent on the stability of the supramolecular complex has been related to the H-bond in terms of its length. The predicted length of the H-bond has been established by the theoretical calculations using the same method, and the results have been tabulated in Table 2. The electronic existence of the attached group had a major impact on the order of the H-bond complex as shown in the table. The electron-releasing MeO and Me groups showed the least strength; however, the electron-withdrawing NO_2_ enhances the H-bonding by decreasing its length. This could be explained in terms of the availability of the lone pair of the electrons in the nitrogen atom of the pyridine moiety, and releasing groups increase the electron donation of the nitrogen atom of the pyridine moiety.

**TABLE 2:** Predicted H-bond length by DFT at B3LYP 6-311G (d,*p*) basis set for (A–F)C8.

Parameter	A/C8	B/C8		C/C8	D/C8	E/C8	F/C8
Polar group	OCH_3_	CH_3_		Cl	Br	F	NO_2_
H-bond length (Ǻ)	1.60990		1.61146	1.63020	1.62782	1.62641	1.64674

### Frontier Molecular Orbitals and Polarizability

**FIGURE 7:**
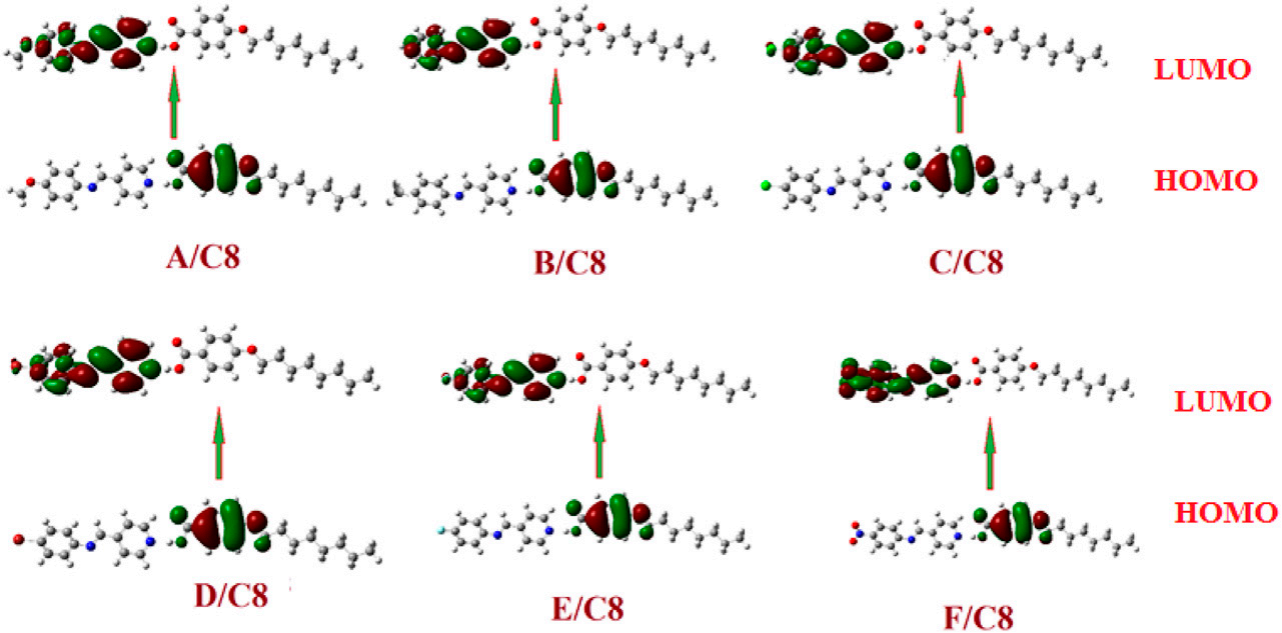
The estimated plots for frontier molecular orbitals of the prepared supramolecular complexes of the octyloxybenzoic acid C8 [(A–F)C8].

The predicted plots of frontier molecular orbitals’ HOMO (highest occupied) and LUMO (lowest unoccupied) of the prepared compounds, (A–F)C8, are shown in Table 3 and Figure 7. Figure 7 shows that the electron densities of the sites involved in the formation of the LUMOs are centered on the pyridyl part as do the aromatic rings of alkoxy benzoic acid in the formation of HOMOs. The polar groups, on the other hand, had no impact on the position of the electron densities of the FMOs. However, the existence of the polar groups had a major effect on the FMOs’ frontier energy gab. The attachment of the polar groups affects the levels of the frontier molecular orbitals. However, the observed effect was observed for the nitro derivative, F/C8. The attachment of the electron-withdrawing NO_2_ group decreases the levels of the HOMO and LUMO with respect to the other groups. On the other hand, the extra-conjugated nitro group leads to a decrement in the energy gab. The nitro group raises the coplanarity and consequently extra conjugation of the aromatic rings and decrements of the FMOs energy gap were observed.

**TABLE 3:** FMO energies (eV) and its levels of the prepared supramolecular complexes of the octyloxybenzoic acid C8 [(A-F)C8].

Compound	HOMO	LUMO	ΔE
A/C8	−6.06	−2.50	3.56
B/C8	−6.10	−2.61	3.49
C/C8	−6.15	−2.82	3.33
D/C8	−6.14	−2.79	3.35
E/C8	−6.14	−2.76	3.38
F/C8	−6.23	−3.46	2.77

**TABLE 4:** Mesomorphic parameters, dipole moment, μ, polarizability, α, and aspect ratio of the prepared supramolecular complexes of the octyloxybenzoic acid C8 [(A-F)C8].

Compound	Group	ΔT_N_	ΔT_N_	ΔT (mesomorphic range)	T_C_ (mesomorphic stability)	Dipole moment, μ	Dimension Ǻ		Aspect ratio (L/D)
							Width (D)	Length (L)	
A/C8	OCH_3_	0	38.0	38.0	128.4	3.89	4.94	34.55	6.99
B/C8	CH_3_	0	19.2	19.2	94.6	4.19	5.04	32.37	6.42
C/C8	Cl	0	45.1	45.1	133.8	1.95	5.06	33.50	6.62
D/C8	Br	0	39.2	39.2	134.5	2.12	5.12	33.79	6.60
E/C8	F	21.7	49.9	71.6	147.1	2.12	5.04	32.60	6.47
F/C8	NO_2_	0	48.5	48.5	124.0	3.62	5.14	33.50	6.52

**FIGURE 8:**
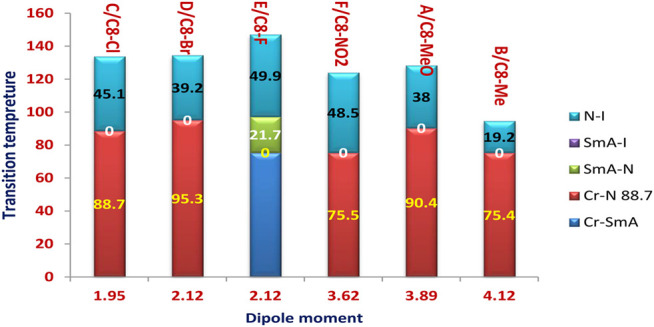
Dependence of the transition temperature on the calculated dipole moment of the analogous series of the C8 chain length.

The transition temperature of the analogous sequence of the C8 chain length is shown in Figure 8 as a function of the measured dipole moment. Except for the compact F atom and the highest electron-withdrawing NO_2_ group, it is obvious that a higher dipole moment reduces the nematic range. This finding may be clarified by the enhancement of parallel intermolecular interactions, which allows for a high degree of ordering while reducing the development of less organized nematic phases. On the other hand, the smectic mesophase enhancement may be explained by the high dipole moment associated with the small compact F group. Although there is little difference in the measured dipole moment between methyl and methoxy, there is a significant difference in nematic mesophase temperatures of 38.0 and 19.2°C, respectively. This may be due to the MeO group's resonance effect, which can increase the π-loop and, as a result, the side–side interaction.

**FIGURE 9:**
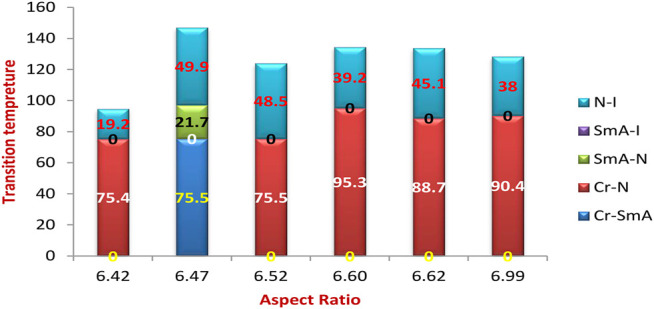
Dependence of the transition temperature on the calculated aspect ratio of the analogous series of the C8 chain length.

The range of the mesophases is somewhat influenced by the aspect ratio of the prepared complexes, as shown in Figure 9, the relation between the transition temperature of the analogous series of the C8 chain length, and the measured aspect ratio. As the aspect ratio increases, Me = 6.42 and MeO = 6.99, the nematic mesophase increases to 19.2 and 38.0°C, respectively. Similarly, the little increment of the aspect ratio of the halide derivatives, 6.62 of the Cl derivative compared with the 6.60 of the Br derivative, enhances the nematic range to 45.1°C instead of 39.2°C of the smaller aspect ratio Cl derivative. However, this situation is not congruent with the MeO derivative compared with the NO_2_ one. This may be explained by the nitro derivative having a higher degree of π-stacking than the methoxy one.

### Molecular Electrostatic Potential

**FIGURE 10:**
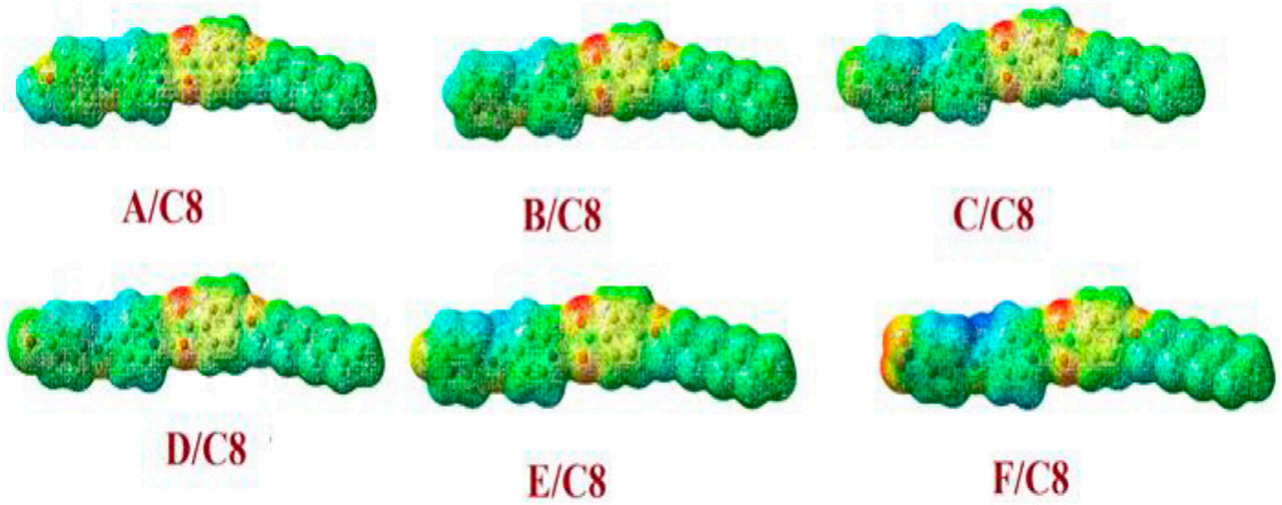
Molecular electrostatic potentials (MEPs) for the prepared supramolecular complexes of the octyloxybenzoic acid C8 [(A–F)C8].

According to molecular electrostatic potential (MEP), the charge distribution map for the prepared supramolecular complexes of the octyloxybenzoic acid C8, [(A–F)C8], was determined using the same method of calculation on the same basis sets (Figure 10). The negatively charged atomic sites (the red region) were thought to be concentrated in the hydrogen-bonded portion of supramolecular complexes. However, regardless of the composition of the polar attached groups, the alkoxy chain moieties were expected to have the least negatively charged atomic sites (blue regions). The formation of the enhanced mesophase of liquid crystals could be clarified by the results of the charges’ distribution mapping.

## Conclusion

New supramolecular three-ring Schiff base liquid crystal complexes were prepared and investigated. The mesomorphic characteristics of the samples showed that all samples exhibit enantiotropic mesophases. Both the polar compact groups’ polarity and alkoxy chain lengths contribute strongly to mesomorphic characteristics and thermal stabilities of the mesophases. Surprisingly, the observed values associated with the crystalline mesomorphic transitions are considerably small (∼2.2–12.5 kJ/mol), indicating a weak hydrogen bonding in the crystalline solid compared to the literature values for crystalline mesomorphic transitions (∼20–100 kJ/mol). However, the enthalpy changes corresponding to the mesomorphic–isotropic transitions vary from 0.9 to 13.9, depending on the polarity nature of para-attached groups. Most complexes showed monomorphic behavior with variant nematic ranges. However, the incorporation of the compact F atom with highest electron-withdrawing inductive effect enhances dimorphic mesophases and nematic and smectic A. The enhanced smectic mesophase was rationalized to the high parallel interaction that could be promoted by the attachment of the F atom, leading to a longer total mesophase range of 71.6°C. The results proved that the nematic mesophase range decreases as the length of the terminal alkoxy chain increases with enhancement of a smectic mesophase appearance for complexes having an alkoxy chain length of 16 carbon atoms. The decrement of the nematic range could be attributed to the higher terminal aggregation of the alkoxy chain which facilitates higher backing of the molecules. Furthermore, the results were discussed in the frame of the DFT calculations. Dependence of the transition temperature on the dipole moment was reported. A minor increment of the aspect ratio of the halide derivatives enhances the nematic range to 45.1°C instead of 39.2°C of the smaller aspect ratio Cl derivative. The situation is inverted while comparing the MeO and the NO_2_ derivatives. This could be understood in terms of the higher degree of π–π stacking exerted by the nitro derivative compared to that of the methoxy one.

## Data Availability

The original contributions presented in the study are included in the article. Further inquiries can be directed to the corresponding author.
